# Role of oxidants in enhancing dewaterability of anaerobically digested sludge through Fe (II) activated oxidation processes: hydrogen peroxide *versus* persulfate

**DOI:** 10.1038/srep24800

**Published:** 2016-04-25

**Authors:** Kang Song, Xu Zhou, Yiqi Liu, Yanyan Gong, Beibei Zhou, Dongbo Wang, Qilin Wang

**Affiliations:** 1 Advanced Water Management Centre, The University of Queensland, St Lucia, Queensland 4072, Australia; 2 Institute of Engineering, Tokyo University of Agriculture and Technology, Tokyo 184-8588, Japan; 3 Harbin Institute of Technology Shenzhen Graduate School, Shenzhen 518055, China; 4 School of Automation Science & Engineering, South China University of Technology, Guangdong 510640, China; 5 Key Laboratory of Pollution Processes and Environmental Criteria (Ministry of Education), Tianjin Engineering Center of Environmental Diagnosis and Contamination Remediation, College of Environmental Science and Engineering, Nankai University, Tianjin 300071, China

## Abstract

Improving dewaterability of sludge is important for the disposal of sludge in wastewater treatment plants (WWTPs). This study, for the first time, investigated the Fe(II) activated oxidization processes in improving anaerobically digested sludge (ADS) dewaterability. The combination of Fe(II) (0–100 mg/g total solids (TS)) and persulfate (0–1,000 mg/g TS) under neutral pH as well as the combination of Fe(II) (0–100 mg/g TS) and hydrogen peroxide (HP) (0–1,000 mg/g TS) under pH 3.0 were used to examine and compare their effect on the ADS dewaterability enhancement. The highest ADS dewaterability enhancement was attained at 25 mg Fe(II)/g TS and 50 mg HP/g TS, when the CST (CST: the capillary suction time, a sludge dewaterability indicator) was reduced by 95%. In contrast, the highest CST reduction in Fe(II)-persulfate conditioning was 90%, which was obtained at 50 mg Fe(II)/g TS and 250 mg persulfate/g TS. The results showed that Fe(II)-HP conditioning was comparable with Fe(II)-persulfate conditioning in terms of highest CST reduction. Economic analysis suggested that the Fe(II)-HP conditioning was more promising for improving ADS dewaterability compared with Fe(II)-persulfate conditioning, with the saving being up to $65,000 per year in a WWTP with a population equivalent of 100,000.

It is well-known that activated sludge processes are the most commonly used method of wastewater treatment. Despite its high efficiency in removing organic substances, huge amounts of waste activated sludge (WAS) are also produced, which causes serious environmental problems and must be treated and disposed of. However, the cost associated with the WAS treatment and disposal was expensive, which could occupy 30–55% of the total operating costs for a wastewater treatment plant (WWTP)^1^,^2^,^3^,^4^,^5^.

The sludge treatment and disposal procedure usually consists of thickening, stabilization, conditioning and dewatering^1^. Sludge conditioning aimed to improve sludge dewaterability, thus facilitating the removal of water during the subsequent dewatering process. The water contained in sludge could be classified as free water and bound water. The bound water is coupled with sludge by means of capillary forces or chemical bounds, which is difficult to separate. On the contrary, the free water is loosely coupled with sludge structure and therefore is capable of being removed easily during the dewatering process. After the conditioning process, the bound water could be transformed into free water^6^,^7^,^8^,^9^,^10^.

Nowadays, a number of techniques have been developed for WAS conditioning, such as advanced oxidization, acid/alkaline, freezing/thawing, heating, and physical treatment^6^,^7^,^8^,^9^,^10^,^11^,^12^. Among them, classic Fenton’s reaction (a typical advanced oxidization method) is promising as a result of its effectiveness in improving WAS dewaterability together with its environment-friendly characteristics^13^,^14^,^15^,^16^. The classic Fenton’s reaction consists of many chain reactions between ferrous (Fe(II)) and hydrogen peroxide (HP) in acidic condition (Eqs (1, 2, 3, 4, 5, 6, 7))^17^. These reactions generate huge amounts of hydroxyl radicals (HO·), which is a highly reactive oxidizing agent^18^. The hydroxyl radicals could oxidize sludge structure. These reactions could facilitate the conditioning of sludge and improve the WAS dewaterability^19^,^20^.


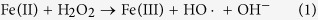



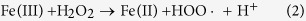



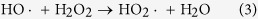



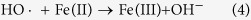



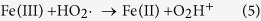



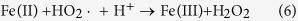



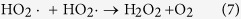


Recently, Zhen *et al*.^10^ reported an innovative method to enhance dewaterability of WAS by Fe(II)-activated persulfate oxidation. They presented that Fe(II)-persulfate oxidation was capable of enhancing WAS dewaterability. In the Fe(II)-persulfate conditioning, through the reduction of persulfate anion (S_2_O_8_^2−^) by Fe(II), the sulfate radical (˙SO_4_^−^) is capable of being produced (Eq. 8)^21^, which has a high oxidation potential^10^.


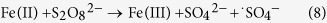


In many WWTPs, anaerobic digestion process is widely utilized to reduce WAS and generate methane before dewatering^1^,^22^,^23^,^24^. In this process, anaerobically digested sludge (ADS) are substantially produced. Different from the average WAS, the ADS is more difficult to dewater, and the current conditioning methods are unsatisfactory^25^. Therefore, high-efficiency conditioning methods for ADS are also needed.

In this study, we investigated and compared the role of HP and persulfate in enhancing dewaterability of ADS through Fe(II) activated oxidation processes for the first time. Capillary suction time (CST) was used as the indicator of ADS dewaterability, which represents the time used for completing the sludge filtration process. Soluble chemical oxygen demand (SCOD) and total iron concentrations in ADS were also measured. Moreover, economic analysis was performed to compare the economic potentials of the Fe(II)-HP and Fe(II)-persulfate conditioning methods.

## Results

### Effect of Fe(II) concentration on ADS dewaterability

**Figure 1:**
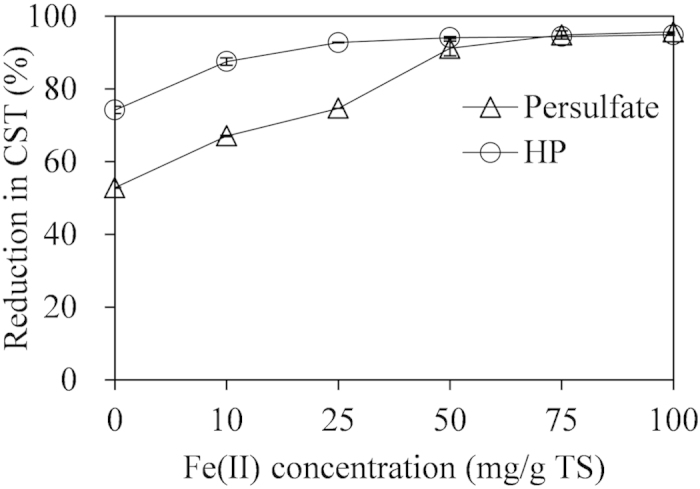
Effect of Fe(II) concentration on ADS dewaterability. Persulfate and HP concentrations were both at 250 mg/g TS. The CST of original ADS without any conditioning was used as the reference.

The effect of Fe(II) concentrations on the ADS dewaterability could been seen in Fig. 1. The ADS dewaterability was enhanced significantly when Fe(II)-persulfate or Fe(II)-HP were added. For the Fe(II)-persulfate conditioning, the reduction percentage of CST rose from 55% to 90% (p < 0.05) while the Fe(II) concentration increased from 0 to 50 mg/g TS at a concentration of 250 mg persulfate/g TS under neutral pH (around 7.6). For Fe(II)-HP conditioning, the reduction percentage of CST increased from 75% to 93% (p < 0.05) when the Fe(II) concentration rose from 0 to 25 mg/g TS at a concentration of 250 mg HP/g TS at pH 3.0. During the processes, the increased concentration of Fe(II) led to the production of more hydroxyl radicals (HO·) (Eq. 1) or persulfate radicals (˙SO_4_^−^) (Eq. 8), which oxidized sludge flocs to a greater extent. Afterwards, bound water was transformed into free water and thus improved the ADS dewaterbaility^26^.

Nevertheless, the dewaterability of ADS was not further improved (p > 0.05) while the Fe(II) concentration increased from 50 to 100 mg/g TS in Fe(II)-persulfate conditioning and from 25 to 100 mg/g TS in Fe(II)-HP conditioning. This might be because that excess addition of Fe(II) could react with sulfate radicals (Eq. 9) and thus consumed the sulfate radicals in Fe(II)-persulfate conditioning. Similarly, in Fe(II)-HP conditioning, excess Fe(II) could also deplete the hydroxyl radicals (Eq. 4). The results indicated that excess addition of Fe(II) (>50 mg/g TS in Fe(II)-persulfate conditioning and >25 mg/g TS in Fe(II)-HP conditioning) could not lead to the corresponding improvement of ADS dewaterability.


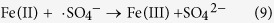


It should be noteworthy that the reduction percentage of CST (approximately 90%) in this study was substantially higher than those (approximately 50%) reported in the previous studies, in which similar oxidants were used to enhance the WAS dewaterability^27^,^28^. The reason might be due to the diverse characteristics between WAS and ADS. For instance, they have different shear sensitivity, structural properties, chemical compositions (e.g. concentration of polysaccharide and protein) of intracellular substances and extracellular polymeric substances^29^,^30^, and thus different filterability and dewaterability performance^31^,^32^,^33^. This is also why this study was carried out.

**Figure 2:**
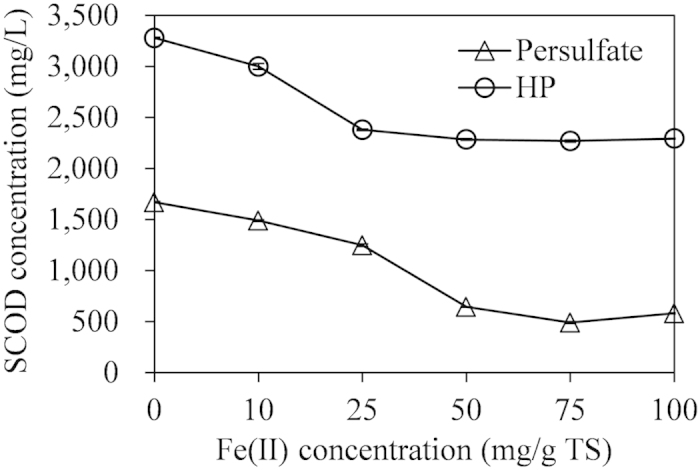
Effect of Fe(II) concentration on SCOD concentration. Persulfate and HP concentrations were both at 250 mg/g TS.

In addition, it was found that the increased Fe(II) concentration from 0 to 50 mg/g TS resulted in the decreased SCOD concentrations (from 1,670 to 640 mg/L) in Fe(II)-persulfate conditioning. Likewise, SCOD concentrations decreased from 3,280 to 2,380 mg/L while Fe(II) concentration increased from 0 to 25 mg/g TS in Fe(II)-HP conditioning (see Fig. 2). This might be due to the increased oxidization capacity of the oxidization processes^34^,^35^, which was consistent with the CST result. However, further increase of Fe(II) failed to induce an decrease in SCOD, which might be due to the consumption of free radicals, as discussed in the previous sections.

### Effect of persulfate/HP concentration on ADS dewaterability

**Figure 3:**
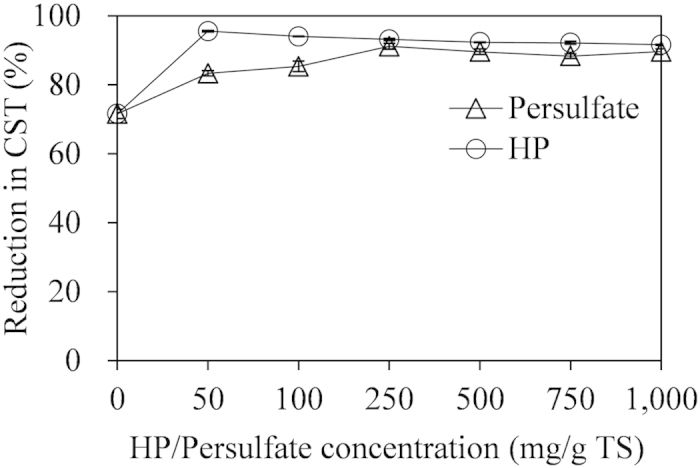
Effect of HP or persulfate concentration on ADS dewaterability. Fe(II) concentration in persulfate and HP conditioning sets were 50 and 25 mg/g TS, respectively. The CST of original ADS without any conditioning was used as the reference.

Based on Fig. 1, 50 mg Fe(II)/g TS was sufficient in enhancing ADS dewaterability in the Fe(II)-persulfate conditioning. Thus, the effect of persulfate concentration on ADS dewaterability was explored at 50 mg Fe(II)/g TS. Figure 3 showed that the CST reduction percentage rose from 70% to 90% (p < 0.05) when the concentration of persulfate increased from 0 to 250 mg/g TS. However, when the concentration of persulfate further rose from 250 mg/g TS to 1,000 mg/g TS, the ADS dewaterability did not increase any more. The phenomenon was in accordance with the previous study^26^,^36^, which reported that adding excessive persulfate could not accelerate the advanced oxidization processes.

For the Fe(II)-HP conditioning, it was found that 25 mg Fe(II)/g TS was enough in improving ADS dewaterability. Therefore, the impact of HP concentration on ADS dewaterability was investigated at 25 mg Fe(II)/g TS. Figure 3 showed that the reduction percentage of CST increased from 70% to 95% (p < 0.05) when the concentration of HP rose from 0 to 50 mg/g TS. However, when the concentration of HP further increased to 1,000 mg/g TS, the ADS dewaterability declined slightly (p < 0.05). It was due to the fact that the addition of excess HP could consume hydroxyl radicals (Eq. 3), thereby impacting the oxidization processes negatively.

## Discussion

### Comparison of Fe(II)-persulfate conditioning and Fe(II)-HP conditioning on ADS dewaterability

**Figure 4:**
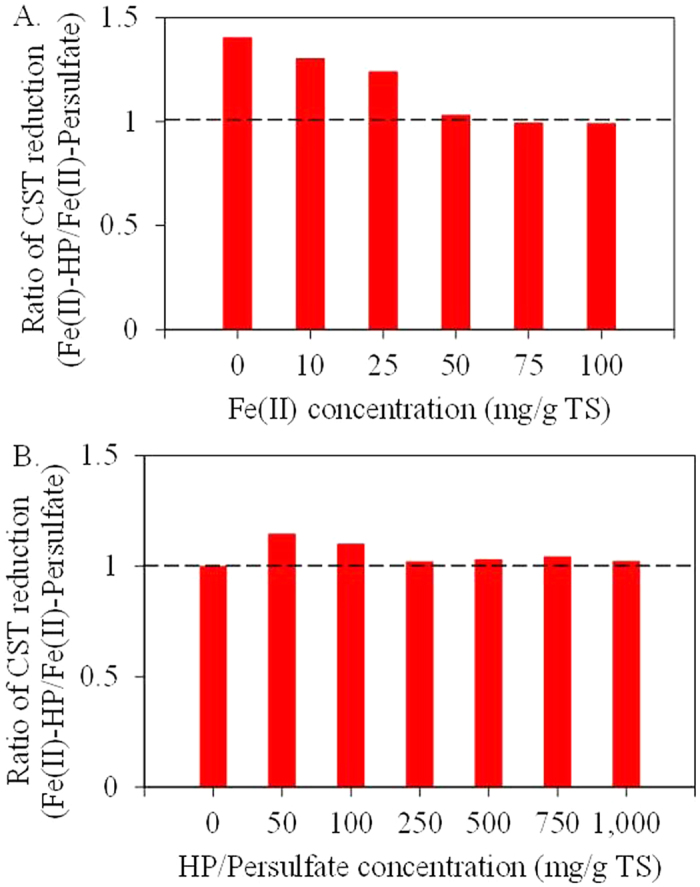
Ratio of CST reduction percentage of Fe(II)-HP to Fe(II)-persulfate conditionings (Calculation method: CST reduction percentage of Fe(II)-HP conditioning divided by CST reduction percentage of Fe(II)-persulfate conditioning). (**A**) Effect of Fe(II) concentration on ADS dewaterability. Persulfate and HP concentrations were both at 250 mg/g TS. (**B**) Effect of oxidant (i.e. persulfate or HP) concentration on ADS dewaterability. Fe(II) concentrations in persulfate and HP conditioning sets were 50 and 25 mg/g TS, respectively.

This study demonstrated that both Fe(II)-persulfate conditioning and Fe(II)-HP conditioning were effective in improving ADS dewaterability. In order to compare both methods, the ratios of CST reduction percentages of Fe(II)-HP to Fe(II)-persulfate conditionings were calculated and shown in Fig. 4. Figure 4A was to compare the effect of Fe(II) concentration (0–100 mg/g TS) on ADS dewaterability when both persulfate and HP concentrations were at 250 mg/g TS. Figure 4A showed that the CST reduction percentages of Fe(II)-HP conditioning were higher (1.2–1.4 times) than those of Fe(II)-persulfate conditioning (p < 0.05) while the Fe(II) concentration was less than 50 mg/g TS. However, the CST reduction percentages of Fe(II)-HP conditioning were comparable with those of Fe(II)-persulfate conditioning (p > 0.05) when the Fe(II) concentration was higher than 50 mg/g TS. Meanwhile, it was interesting that when there was no Fe(II) addition in the system, the ratio of CST reduction percentage of Fe(II)-HP to Fe(II)-persulfate conditionings was the highest (i.e. 1.4). This is likely due to the fact that HP could react with the indigenous iron (i.e. iron in sludge) and generated hydroxyl radicals under acidic conditions^37^. The results implied that HP might be more suitable for sludge conditioning compared with persulfate, especially when the indigenous iron concentration in ADS was high.

Figure 4B was to compare the effect of persulfate or HP concentration (0–1,000 mg/g TS) on ADS dewaterability when the Fe(II) concentration in persulfate and HP conditioning sets were 50 and 25 mg/g TS, respectively. The results showed that the reduction percentages of CST in Fe(II)-HP conditioning were comparable with those of Fe(II)-persulfate conditioning (p > 0.05) in all persulfate and HP concentrations except that when the concentrations of HP or persulfate were at 50 and 100 mg/g TS. The ratio of CST reduction percentage of Fe(II)-HP to Fe(II)-persulfate conditionings attained the maximum value (i.e. 1.2) while the concentration of HP or persulfate was at 50 mg/g TS. It was also noteworthy that the addition of Fe(II) in Fe(II)-HP conditioning (25 mg/g TS) was only half of that (50 mg/g TS) in Fe(II)-persulfate conditioning. Therefore, the Fe(II)-HP conditioning consumed much less chemicals than the Fe(II)-persulfate conditioning.

Furthermore, according to the results attained in batch tests, the highest improvement of ADS dewaterability in the Fe(II)-HP conditioning was obtained at 25 mg Fe(II)/g TS and 50 mg HP/g TS, under which the CST was decreased by 95% (see Fig. 3). However, the largest enhancement of ADS dewaterability in the Fe(II)-persulfate conditioning was obtained at 50 mg Fe(II)/g TS and 250 mg persulfate/g TS, under which the CST was decreased by 90% (see Fig. 3). Regarding the highest CST reduction, Fe(II)-HP conditioning was comparable with Fe(II)-persulfate conditioning. However, in terms of treatment efficiency and economical potential (see the following section for economic analysis), Fe(II)-HP conditioning was more promising for the improvement of ADS dewaterability compared with Fe(II)-persulfate conditioning.

### A potential approach for enhancing ADS dewaterability

In this work, the effects of Fe(II)-persulfate and Fe(II)-HP conditionings on the ADS dewaterability were investigated and compared by laboratory tests for the first time.

In order to compare the economic potential of the Fe(II)-HP conditioning with the Fe(II)-persulfate conditioning process, a desktop scaling-up study was carried out in a WWTP with a population equivalent of 100,000. The results revealed that the Fe(II)-HP conditioning process was capable of saving the cost by up to 60% ($65,000 per year) compared to the Fe(II)-persulfate conditioning process (see Table 1). However, full-scale tests are still needed to better evaluate the economic potentials of the Fe(II)-HP and Fe(II)-persulfate conditioning methods

**Figure 5:**
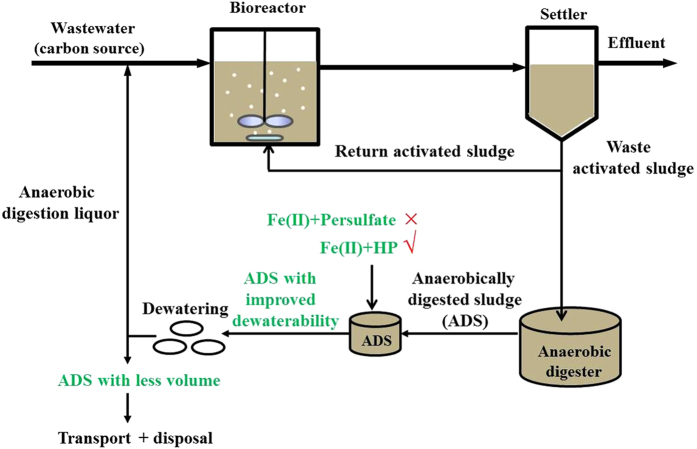
Conceptual diagram of the proposed conditioning approach applied in a typical wastewater treatment plant.

Based on the results, we propose a potential approach for enhancing ADS dewaterability in a WWTP, as shown in Fig. 5. The waste activated sludge (WAS) produced in the wastewater treatment process is firstly fed to the anaerobic digester, in which ADS is generated. ADS is then fed to a small conditioning reactor (i.e. Fe(II)-HP conditioning), in which the ADS dewaterability could be enhanced. Afterwards, the treated ADS is transferred to the dewatering process. Finally, smaller volume of sludge would be produced and disposed of.

In conclusion, a series of batch tests were performed to compare the effects of persulfate and HP on the ADS dewaterability through Fe(II) activated oxidation processes. Compared to the Fe(II)-persulfate conditioning, the Fe(II)-HP conditioning was more effective in enhancing the dewaterability of ADS, with the reduction percentage of capillary suction time being up to 95%. Economic analysis indicated that the Fe(II)-HP conditioning was more economically favorable, the total costs could be saved by 60% ($65,000 per year) in comparison to the Fe(II)-persulfate conditioning process.

## Methods

### Sludge sources and chemicals

The ADS was collected from an anaerobic digester (sludge retention time is 15 days) of a local full-scale WWTP for biological nutrient removal. Its main characteristics are shown in Table 2.

The stock concentration of HP (Ajax Finechem Co.) was 30%. Potassium persulfate (Sigma-Aldrich Co.) and ferrous sulfate (analytical grade, Sigma-Aldrich Co.) were used in this study. 30% sulfuric acid was applied to adjust the pH of ADS.

### Batch tests

Four groups of batch tests were conducted to compare the effects of Fe(II)-persulfate and Fe(II)-HP conditioning processes on ADS dewaterability (Table 3). In the Fe(II)-persulfate conditioning tests, two groups of batch tests were designed (Table 3, Sets A). The group I was to investigate the effect of Fe(II) concentration (0–100 mg/g TS, see Table 3) while the concentration of persulfate was kept at 250 mg/g TS. The group II was to investigate the effect of persulfate concentration (0–1,000 mg/g TS, see Table 3) while the concentration of Fe(II) was kept as 50 mg/g TS. Similarly, in the Fe(II)-HP conditioning tests, two groups of batch tests were also designed (Table 3, Sets B). The group III was to investigate the effect of Fe(II) concentration (0–100 mg/g TS, see Table 3) while the concentration of HP was kept at 250 mg/g TS. The group IV was to elucidate the effect of HP concentration (0–1,000 mg/g TS, see Table 3) while the concentration of Fe(II) was kept at 25 mg/g TS. All the tests were done in duplicate.

In every test, 50 ml of ADS was added to a 150 mL flask. pH of the ADS was not adjusted in the Fe(II)-persulfate conditioning and was observed at around 7.6, whereas pH of the ADS was adjusted to 3.0 by adding sulfuric acid (30%) in the Fe(II)-HP conditioning. Different concentrations of persulfate/HP and Fe(II) were then added to the flasks according to Table 3. Afterwards, the flasks were placed on an orbital shaker for 0.5 h at 150 rpm. The CST and SCOD were then measured according to the approaches detailed in the next Section.

### Analytical procedure and data analysis

The dewaterability of ADS was measured by a capillary suction timer (Trition-WPRL, Type 304). CST is normally used to indicate the dewaterability of sludge^38^, which could measure the time required for sludge to finish the filtration process. The sludge was added to the stainless-steel funnel above the filter paper in the filtration process. After that, the equipment determined the required time of the water in sludge permeated the filter paper.

Iron concentration in the original ADS was determined via Inductive Coupled Plasma connects with Optical Emission Spectroscopy instrument (ICP-OES). The ADS samples were first digested with 70% nitric acid (HNO_3_) for 15 min before performing the measurement. TS, VS, TCOD (Total Chemical Oxygen Demand) and SCOD concentrations were determined based on the standard methods^39^.

The enhancement of ADS dewaterability was assessed via the CST reduction percentage R (%), which is calculated as follows:


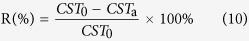


where CST_0_ represents the CST of the original ADS (s); CST_a_ represents the CST of the ADS after conditioning (s).

## Additional Information

**How to cite this article**: Song, K. *et al*. Role of oxidants in enhancing dewaterability of anaerobically digested sludge through Fe(II) activated oxidation processes: hydrogen peroxide *versus* persulfate. *Sci. Rep.* **6**, 24800; doi: 10.1038/srep24800 (2016).

## Figures and Tables

**Table 1:** Economic analysis and comparison of the Fe(II)-HP and Fe(II)-persulfate conditioning processes for the ADS dewaterability enhancement in an assumed WWTP with a population equivalent of 100,000.

**General parameter**		**Values**
Size of the WWTP (Population equivalent - PE)		100,000
Size of the WWTP (m^3^ wastewater/d)		25,000
Influent Chemical Oxygen Demand (COD) (mg/L)		600^a^
Influent Biochemical Oxygen Demand (BOD) (mg/L)		320^a^
Influent Total Kjeldahl Nitrogen (TKN) (mg N/L)		55^a^
Influent ammonium nitrogen (mg N/L)		35^a^
Influent total suspended solids (mg/L)		200^a^
Decay coefficient of heterotrophic biomass (d^−1^)		0.2^b^
Decay coefficient of nitrifying biomass (d^−1^)		0.1^b^
Yield coefficient of heterotrophic biomass (g COD/g COD)		0.625^b^
Yield coefficient of nitrifying biomass (g COD/g N)		0.24^b^
Fraction of inert COD generated in biomass decay (g COD/g COD)		0.2^b^
Sludge retention time in the bioreactor of the WWTP (d)		12^a^
Mixed liquor suspended solid concentration in the bioreactor (mg/L)		4,000^a^
Mixed liquor volatile suspended solid concentration in the bioreactor (mg/L)		3,200^a^
Hydraulic retention time of the anaerobic digester (d)		20^b^
Degradation of waste activated sludge in anaerobic digester (on a dry VS basis)		35%^a^
Price of ferrous sulfate ($/tonne)		100^c^
Price of 50% hydrogen peroxide ($/tonne)		450^c^
Price of sulfuric acid ($/tonne)		250^c^
Price of potassium persulfate ($/tonne)		600^c^
Fe(II)-HP conditioning	ADS subject to conditioning (dry tonne/y)	560
	Ferrous sulfate concentration (mg Fe(II)/g TS)	25
	Hydrogen peroxide (mg HP/g TS)	50
	Reduction percentage of CST	90%
	Ferrous sulfate cost ($/y)	3,800
	Hydrogen peroxide cost ($/y)	25,200
	Sulfuric acid cost ($/y)	12,300
	**Total cost ($/y)**	**41,300**
Fe(II)-persulfate conditioning	AD subject to conditioning (dry tonne/y)	560
	Ferrous sulfate concentration (mg Fe(II)/g TS)	50
	Potassium persulfate (mg perfulfate/g TS)	250
	Reduction percentage of CST	90%
	Ferrous sulfate cost ($/y)	7,600
	Potassium persulfate cost ($/y)	99,000
	**Total cost ($/y)**	**106,600**
	Saving with Fe(II)-HP conditioning (compared toFe(II)-persulfate conditioning) ($/year)	65,000

^a^Personal communication with industry partners.

^b^Refer to Metcalf and Eddy, (2003).

^c^http://www.alibaba.com/.

**Table 2:** Main characteristics of ADS.

Iron (mg/L)	282
Total solids (TS, g/L)	28.3 ± 0.4
Volatile solids (VS, g/L)	20.1 ± 0.2
Solid content (%)	2.83 ± 0.04
Moisture content (%)	97.17 ± 0.04
TCOD (g/L)	23.1 ± 0.2
SCOD (g/L)	710 ± 5
CST (s)	188.6 ± 0.7
pH	7.6

**Table 3:** Experiment conditions applied in the batch tests.

**Sets A**	**Fe(II)-persulfate conditioning**^a^		**Sets B**	**Fe(II)-HP conditioning**^b^	
	**Persulfate concentration(mg/g TS)**	**Fe(II) concentration(mg/g TS)**		**HP concentration(mg/g TS)**	**Fe(II) concentration(mg/g TS)**
I. Effect of Fe(II)concentration	250	0	III. Effectof Fe(II)concentration	250	0
	250	10		250	10
	250	25		250	25
	250	50		250	50
	250	75		250	75
	250	100		250	100
II. Effect of persulfateconcentration	0	50	IV. Effectof HPconcentration	0	25
	50	50		50	25
	100	50		100	25
	250	50		250	25
	500	50		500	25
	750	50		750	25
	1,000	50		1,000	25

^a^pH was not adjusted and was observed at around 7.6.

^b^pH was adjusted to 3.0 by adding sulfuric acid (30%).
